# Cancer Screening Interventions in Indigenous Populations: A Rapid Review

**DOI:** 10.3390/curroncol28030161

**Published:** 2021-05-06

**Authors:** Janell Bryant, Kara Patterson, Marcus Vaska, Bonnie Chiang, Angeline Letendre, Lea Bill, Huiming Yang, Karen Kopciuk

**Affiliations:** 1 Screening Programs, Provincial Population and Public Health, Alberta Health Services, 2210-2 Street SW, Calgary, AB T2S 3C3, Canada; Janell.Bryant@albertahealthservices.ca (J.B.); Kara.Patterson@albertahealthservices.ca (K.P.); Bonnie.Chiang@albertahealthservices.ca (B.C.); Huiming.Yang@albertahealthservices.ca (H.Y.); 2 Knowledge Resource Service, Alberta Health Services, 1331-29 Street NW, Calgary, AB T2N 4N2, Canada; Marcus.Vaska@albertahealthservices.ca; 3 Alberta Cancer Prevention Legacy Fund, Population, Public and Indigenous Health, Alberta Health Services, 102 Anderson Hall, 10959 102 ST NW, Edmonton, AB T5H 3V9, Canada; Angeline.Letendre@albertahealthservices.ca; 4 Alberta First Nations Information Governance Centre, P.O. Box 1459, Siksika, AB T0J 3W0, Canada; lea.bill@afnigc.ca; 5 Department of Community Health Sciences, University of Calgary, 2500 University Dr NW, Calgary, AB T2N 1N4, Canada; 6 Departments of Oncology and Mathematics and Statistics, University of Calgary, 2500 University Dr NW, Calgary, AB T2N 1N4, Canada; 7 Cancer Epidemiology and Prevention Research, Cancer Care Alberta, Alberta Health Services, 2210-2 Street SW, Calgary, AB T2S 3C3, Canada

**Keywords:** attitudes, cancer screening, community-based trial, Indigenous people, intentions, interventions, knowledge, Plan-Do-Study-Act cycles trial, randomized control trial

## Abstract

Cancer screening is an important component of a cancer control strategy. Indigenous people in Canada have higher incidence rates for many types of cancer, including those that can be detected early or prevented through organized screening programs. Increased participation and retention in cancer screening is critical to improved population health outcomes amongst Indigenous people. This rapid review evaluates cancer screening interventions published in the last six years. Included studies demonstrated increased participation in breast, colorectal, or cervical cancer screening programs in Indigenous populations or showed promise of increased participation based on the factors that influence people’s screening practices, such as knowledge, attitude, or intent to screen. The Preferred Reporting Items for Systematic Reviews guided the search strategy. The review identified 85 articles with 12 meeting the specified criteria: seven studies reported an increase in cancer screening participation and five studies reported improved knowledge, attitude, or intent to screen. The use of multiple culturally appropriate strategies in co-designed studies were the most effective. This review will be used to inform First Nations (FN) populations and Screening Programs in Alberta of potential strategies to address disparities identified through a recent data analysis comparing cancer screening and outcomes between FN and non-FN people.

## 1. Background

### 1.1. Disparities in Cancer Screening Among Indigenous Populations

Cancer is the third most common cause of death among First Nations (FN) people living in Alberta. Breast, lung, colorectal, and prostate cancer were the most common types of cancers diagnosed among this population from 1997 to 2010. The incidence of some cancers in FN populations, such as cancers of the cervix, stomach, liver, and intrahepatic bile duct is higher than non-FN people in Alberta [1]. In addition, FN people have significantly lower cancer survival rates than non-FN people [2]. 

Screening for breast, colorectal, and cervical cancer may reduce disparities in cancer outcomes by detecting these cancers early at the most treatable stage or prevent cancer from developing. However, many Indigenous Canadians—FN, Inuit, and Métis (FNIM)—experience significant barriers to cancer screening and prevention programs compared with non-Indigenous Canadians [3]. Some Canadian evidence indicates that the participation by Indigenous people in organized cancer screening programs is lower than for non-Indigenous people [4]. In 2015, total cancer screening rates were higher for all of Alberta compared to its rural and remote North Zone for breast (56.7% vs. 48.7%), cervical (62% vs. 56.9%), and colorectal (39.2% vs. 36.1%) cancer screening [5]. The North Zone includes most of the 24 FNs from Treaty 8, or almost half of the 45 Nations in Alberta [6]. Some evidence indicated lower screening rates among FN people than non-FN people in the North Zone.

There are no identifiers for race or ethnicity in provincial cancer registries and most health administrative databases in Canada, making a complete understanding of Indigenous cancer screening practices difficult [3]. To better understand the screening practices of FN people in Alberta’s three provincial cancer screening programs (breast, cervical, colorectal), a recent study assessed cancer screening utilization and outcomes among FN people in Alberta in partnership with the Alberta First Nations Information Governance Centre (AFNIGC). This research identified disparities in all three screening programs but was not designed to explore the reasons or solutions for these disparities. The purpose of this rapid review was to inform future co-planning by FN communities and Screening Programs through the identification of effective and feasible cancer screening interventions that may address disparities in cancer screening participation among FN people in Alberta and beyond.

### 1.2. Increasing Cancer Screening in Indigenous Populations

Worldwide, Indigenous cancer screening rates vary for breast, cervical, and colorectal cancers [4]. Indigenous peoples face unique and complex systemic, cultural, and personal barriers to cancer screening [3]. A commonality among the studies included in this review was the removal of barriers to cancer screening participation. A review by Hutchinson et al. (2018) identified the following barriers to cancer screening among Indigenous people living in Canada:

(1) Attitudes and beliefs about cancer;
(2) Health system challenges;
(3) Lack of trusting relationships with health care providers and health organizations;
(4) Lack of knowledge or awareness about cancer and cancer screening;
(5) Barriers associated with demographics and health determinants;
(6) Impacts of colonialism, discrimination, and/or racism.

In addition, recommendations identified in the FN Health Status Report: Alberta Region (2011–2012) for increasing cancer screening participation included aligning culturally sensitive health services with FN cultures, making greater use of FN patient navigators, and increasing access to screening initiatives in remote and rural areas.

## 2. Methods

The search and review of relevant literature was guided by the following research questions:

Internationally, what cancer screening interventions published in the last six years report:

(1) Increased breast, colorectal, or cervical cancer screening participation in Indigenous populations?
(2) Promise for increasing breast, colorectal, or cervical cancer screening in Indigenous populations based on process indicators of the outcome (e.g., knowledge, attitude, or intent to screen)?

In total, 85 articles were assessed and 12 were included in this review on the basis of alignment with the research question and inclusion/exclusion criteria. The literature search was expanded to include manual searches of relevant webpages and reference lists of included articles.

### 2.1. Search Strategy

To identify relevant articles, a comprehensive search strategy was developed in consultation with a librarian. Ten databases were searched for articles written in English and published from January 2014 to March 2021. See Table 1 below for the databases searched and relevant search terms.

### 2.2. Selection Strategy

**Figure 1:**
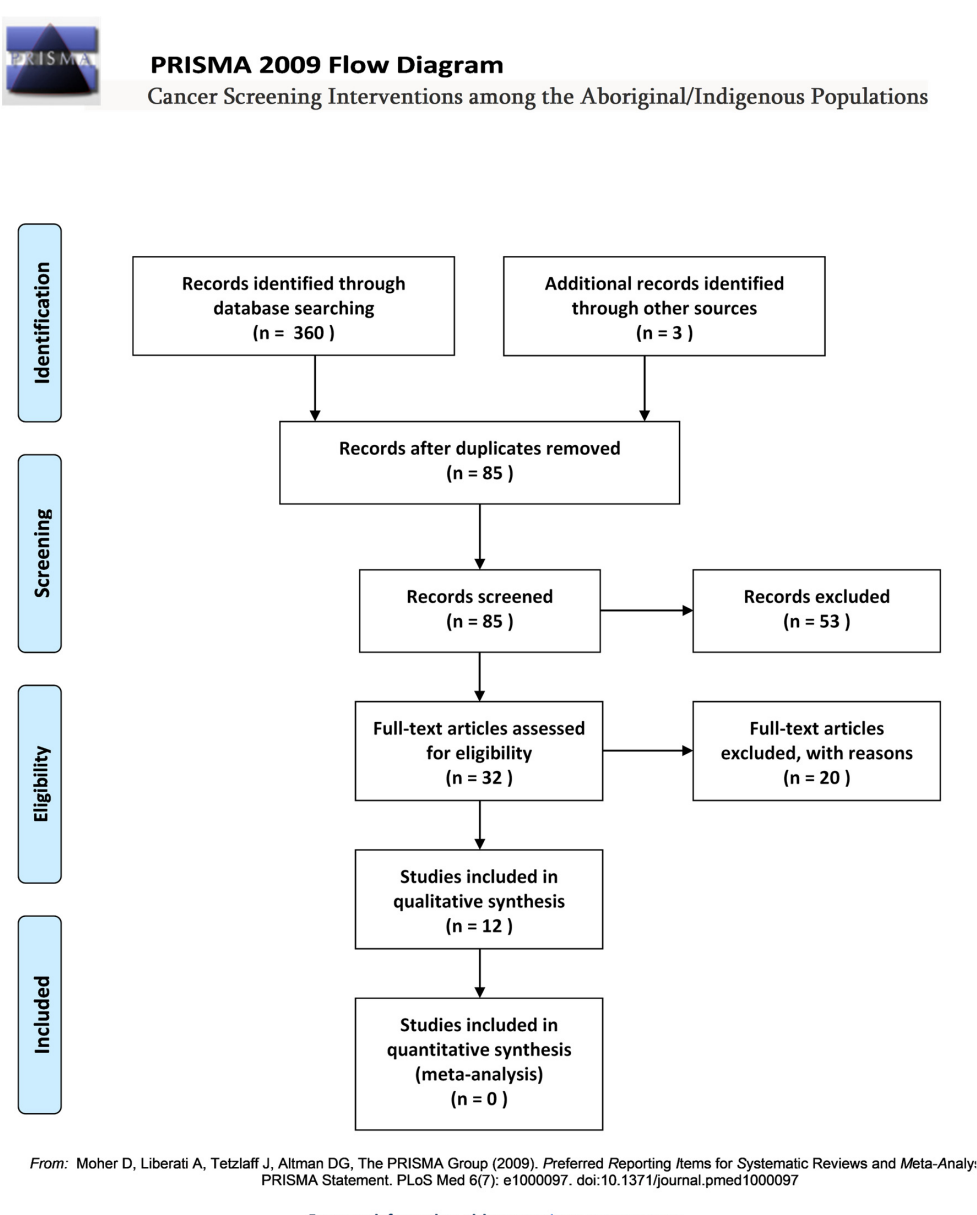
PRISMA flow diagram.

Eighty-five articles were screened first by title and abstract. Thirty-two relevant articles were then read in full and 12 articles were selected for inclusion on the basis of the specified inclusion/exclusion criteria (see Table 2 and PRISMA Flow diagram in Figure 1).

## 3. Interventions That Increased Screening Participation

Seven studies reported an increase in participation for breast, cervical, or colorectal cancer screening in the target population by the end of the intervention (see Table 3). Intervention strategies included various reminder systems, opportunistic screening, mobile screening, Plan-Do-Study-Act (PDSA) cycles, mailed Fecal Immunochemical Test (FIT) kits, and Human papillomavirus (HPV) self-sampling. Target populations for these interventions included Indigenous people in Ontario (Canada), Alberta (Canada), Alaska (USA), New Zealand, and Australia. Outcome measures differed per study and included the rate of screening participation by year, ethnic group, cancer type, and age group.

### 3.1. Randomized Controlled Trials (RCTs)

The following four RCTs led to increased participation for colorectal or cervical cancer screening in the target population by the end of the intervention (see Table 3).

#### 3.1.1. Text-Message Reminders

In the Southcentral Foundation (SCF) healthcare system in Anchorage, Alaska, Muller et al. conducted a two-arm RCT to increase colorectal cancer screening among unscreened Alaskan Natives and American Indian people (AN/AIs) [7]. AN/AIs include people with origins in any of the native peoples of North, South America, and Central America, who have kept their tribal affiliation or community attachment [13]. The two study arms included (1) a text message intervention and (2) a control group receiving standard care. All of those who were eligible for colorectal cancer screening and who signed up to receive text messages were included in the study. Study participants included 2386 AN/AIs aged 40 to 75. Randomization occurred in two waves in November 2013 (*n* = 808) and March 2014 (*n* = 1578).

Following randomization, participants in the intervention group received up to three text messages sent one month apart and those who underwent screening during the intervention stopped receiving texts. Control group participants did not receive any messages during the intervention. Participants in both the intervention and control groups who remained unscreened six months post-intervention received a standard text message.

The wording of text messages for the intervention was developed on the basis of a literature review, key informant interviews with patients (called “customer-owners”) and providers, meetings with tribal leadership, and feedback from focus groups. During the length of the intervention, 181 intervention participants (15.2%) completed colorectal cancer screening, compared with 142 of the control participants (11.9%). The most common method of screening was colonoscopy (>90%).

In the intervention group, there was a 30% significant increase in screening when all age groups were combined (HR = 1.30; 95% CI = 1.04–1.62, *p* = 0.02), a 42% non-significant increase in screening in those aged 50 to 75 (HR = 1.42; 95% CI = 0.97–2.09, *p* = 0.07), and a 24% non-significant increase in screening in those aged 40 to 49 (HR = 1.24; 95% CI = 0.95–1.62, *p* = 0.12). Muller et al. (2017) concluded that text messaging may be an inexpensive and sustainable way to increase screening participation for those who do not require intensive outreach.

#### 3.1.2. Telephone Call Reminders

In New Zealand, Sandiford et al. (2019) conducted a RCT with Māori, Pacific, and Asian individuals who did not return a bowel-testing kit four weeks after receiving it in the mail [8]. Non-respondents were randomized into either (1) a reminder letter and telephone follow up intervention (*n* = 3828) or (2) a standard reminder letter only group (*n* = 3773). Recruitment and randomization started on 9 November 2016 and ended on 3 April 2017. Both intervention and control groups were initially sent reminder letters. A minimum of three telephone calls were made to the intervention group within a four-week period. Participation rates were compared at eight weeks post-randomization.

To help remove cultural barriers, community coordinators with links to the target populations contacted participants and spoke with them in their respective languages. During the calls, community coordinators sought to identify and remove any barriers participants had to screening, such as how to perform the screening test. In the intervention group, there was a 5.2% significant increase in screening participation among Māori (95% CI = 1.8–8.5%), a 3.6% significant increase among Pacific (CI = 0.7–6.4%), and a 0.7% non-significant increase among Asian (CI = −1.1–2.4%) individuals. Māori and Pacific ethnicities lived in areas of higher mean deprivation compared to Asian ethnicities, which was a significant modifier of the effectiveness of the intervention. There was a significant increase in screening among those living in areas of higher deprivation (3.9%; 95% CI = 2.0–5.9%), but not for those living in lower deprivation areas (0.3%; 95% CI = −1.6–2.2%). Thus, a telephone reminder intervention improved colorectal cancer screening participation among Māori and Pacific ethnic groups.

Since live calling was 10 times more expensive than reminder letters, Sandiford and colleagues suggested that it may be more cost-effective to target populations living in areas of high deprivation where direct calling had the greatest impact. They also concluded that it may have been more efficient to delay telephone calls for a few weeks, since a large number of individuals (360 Māori, 349 Pacific, and 1871 Asian) returned the kits within four weeks after receiving them, even without telephone follow-up.

#### 3.1.3. HPV Self-Sampling

In Northland, New Zealand, 931 under-screened/never-screened Māori women, aged 25–69, were included in a cluster randomized controlled trial called *He Tapu Te Whare Tangata* (the sacred house of humankind) between March 2018 and August 2019 [9]. Six primary care clinics were randomly assigned to either the intervention (i.e., HPV self-test) or control (i.e., standard care—a cervical smear by a clinician). Both study arms included a clinic education update on HPV and participant outreach by nurses and kaiāwhina (non-clinical community Māori health workers). Previous research by Adcock et al. [14] found that the majority of Māori women were likely to accept an HPV self-test.

In total, 59.0% (295/500) of Māori women were screened in the intervention arm and 21.8% (94/431) were screened in the control arm. Participants in the intervention arm were 2.8 times more likely to be screened than those in the control arm, after adjusting for age, time since last screen, and deprivation index (95% CI: 2.4–3.1, *p* < 0.0001). Thus, MacDonald and colleagues (2021) concluded that offering HPV self-testing may decrease the amount of under-screened/never-screened Māori women by half. According to the authors, these results may be generalizable to Indigenous peoples with similar barriers in other high-income countries.

#### 3.1.4. Mailing of FIT Kits

Study participants included 1288 AI/AN people, aged 50–75, who were not up to date with colorectal screening when the study began and had no history of colorectal cancer or total colectomy [10]. Participants were recruited from three southwestern United States tribal health care facilities and randomly assigned to one of three study groups, including: (1) usual care (i.e., receiving a FIT kit from a clinic during a regular visit if recommended by a doctor), (2) FIT kit mailing, (3) FIT kit mailing with follow-up outreach by phone and/or home visit from an American Indian Community Health Representative (CHR) if the completed kit was not returned within four weeks of mailing. The intervention period for all study groups was April to November 2014.

In total, 12.8% (165/1288) returned a completed FIT kit. Of those who received usual care (group 1), 6.4% (36/566) returned a completed FIT kit. Among those who received mailed FIT kits without outreach (group 2), 16.9% (61/361) returned a completed FIT kit, a significant increase over usual care (*p* < 0.01). Of those who received mailed FIT kits plus CHR outreach (group 3), 18.8% (68/361) returned the kits, which was a significant increase compared to usual care (*p* < 0.01) but not compared to the mailed FIT kit-only group (*p* = 0.44). The non-significant increase of group 3 compared to group 2 may be in part due to limitations of CHR outreach. That is, during the study period, nearly one in four non-respondents from group 3 did not receive any outreach, due to delays or incorrect contact information.

Among the 165 participants who returned FIT kits, 39 (23.6%) had a positive result and were referred to colonoscopy. Twenty-three (59.0%) followed through with the colonoscopy, of which 12 had polyps and one was diagnosed with colorectal cancer. Thus, the authors concluded that eliminating structural barriers through direct FIT kit mailing may be a useful, population-based strategy to improve colorectal cancer screening rates among AI/AN people.

### 3.2. Pilot Projects

The following two pilot projects reported increased participation for breast, colorectal, and cervical cancer screening in the target population by the end of the intervention (see Table 3).

#### 3.2.1. Opportunistic Screening Pilot

Chow et al. (2020) implemented a pilot project called the Wequedong Lodge Cancer Screening Program (WLCSP) in Northwestern Ontario from October 2013 to November 2016 [11]. Individuals stayed at the lodge while accessing health services in the urban center of Thunder Bay, Ontario. The WLCSP provided cancer screening education and opportunistic breast, cervical, and colorectal cancer screening to those staying at the lodge. This mainly included people from rural and remote FN populations. The program sought to remove geographic, transportation, and cultural barriers by providing accessible, convenient, and culturally sensitive cancer screening services.

A FN liaison was valuable to the program, as they were able to speak with clients in their first language, address language and cultural barriers, and help establish trust. They also provided general administrative support, including recruitment, booking appointments, and follow up. A FN-specific education toolkit was developed and used during appointments, which incorporated storytelling and pictures in a flipbook about cancer screening.

In total, the WLCSP booked 1033 appointments over three years (81% attended; 841/1033). The proportion of eligible clients to participate in screening included: mammogram, 22% (60/275); Pap test, 8% (45/554); and fecal occult blood test, 32% (106/333). The number of clients increased by 62% from 2014–2015 and 68% from 2015–2016. Approximately 9500 adults stayed at Wequedong Lodge each year; thus, it was estimated that 2% (157/9500) attended the program in 2014, 3% (255/9500) in 2015, and 5% (429/9500) in 2016.

One possible limitation of an opportunistic screening program is that it does not typically allow enough time to build trusting relationships with healthcare providers. However, opportunistic screening may provide an alternative way to screen under- or never-screened individuals who would not usually participate in an organized, population-based program.

#### 3.2.2. Mobile Screening Pilot.

The Enhanced Access to Cervical and Colorectal Screening (EACS) program was a two-year pilot project offered in Alberta between 2013 and 2014 [5]. EACS integrated cervical and colorectal cancer screening with the Screen Test mobile mammography program. It sought to improve access to cervical and colorectal cancer screening in rural and remote FN, Métis, and Hutterite populations by removing geographical barriers and increasing awareness through a convenient “one-stop shop”/integration of screening services.

The most remote and underserviced areas were selected to receive the integrated Screen Test-EACS intervention while other areas received only Screen Test (usual practice) mammography services. In total, 8390 women from 44 communities participated in Screen Test mammography services, and 1312 women from 16 communities participated in Screen Test-EACS. Screen Test-EACS significantly increased the uptake of cancer screening compared to clients in the communities with only Screen Test mammography services for cervical (10.1% with Screen Test vs. 27.5% with Screen Test-EACS) and colorectal (10.9% with Screen Test vs. 22.5% with Screen Test-EACS) cancer screening (*p* < 0.0001 for all variables). In addition, Screen Test-EACS led to a significant net increase in prevalence of clients up to date with cervical (52.5% vs. 62.9%) and colorectal (37.3% vs. 48.7%) cancer screening three months after getting a mammogram (significance levels not reported). Alberta Health Services (AHS) Screening Programs is currently in the process of planning a second phase of the integrated screening project.

### 3.3. Translational Research

The following translational research study reported increased cervical cancer screening participation in the target population by the end of the intervention (see Table 3).

#### Plan-Do-Study-Act (PDSA) Cycles.

In Australia, Dorrington and colleagues led an intervention targeting Aboriginal and Torres Strait Islander women within the urban Aboriginal Community Controlled Health Service (ACCHS) [12]. In 2012, five rapid PDSA cycles, each lasting four to five weeks, led to a 40% significant increase in cervical screening (*n* = 217) compared to the average of the previous three years (mean = 170; s.d. = 33.2; *p* = 0.002). This increase was sustained for 10 months of follow up.

The PDSA cycles were conducted using translational research and continuous quality improvement informed by client surveys, a data collection tool, focus groups, and internal research. The core of the research included community and service collaboration and knowledge acquisition from ACCHS clients and staff, internal research, and data. This was done to identify and address local barriers and facilitators to cervical screening. Interventions were planned on the basis of their practicality, likelihood of success, and cultural acceptability. Interventions were implemented during each cycle and included a data collection tool for healthcare providers, promotional materials (i.e., posters, make-up mirrors, and nail files), an afternoon clinic designated for appointments rather than the usual walk-ins, updated reminder letters, recall system review and clean-up, and education of the Social Health Team around prevention. Due to the rapid nature of the PDSA cycles, the impact on cervical screening per cycle was not determined.

A benefit of this model was its transferability to other setting and health issues. Sustainability of the program may be difficult, due to its rapid and intensive nature. However, less intensity is required over longer periods, which may make the cycles more sustainable.

## 4. Interventions That Improved Knowledge, Attitude, or Intention to Screen

Five studies showed promise on the basis of process indicators for increasing cancer screening participation rates (e.g., improved knowledge, attitude, or intent to screen). Intervention strategies included community engagement, peer support, and human papillomavirus (HPV) self-sampling (see Table 4). Target populations for these interventions included Indigenous people from New Zealand and Ontario (Canada), as well as Native Americans from Arizona and Oklahoma (USA), and Indigenous people of Hawaii (USA).

### 4.1. Community-Based Participatory Research

The following two community-based research studies showed promise of increased participation in colorectal and breast cancer screening based on process indicators of this outcome (see Table 4).

#### 4.1.1. Peer-Led Intervention

Cassel et al. (2020) led a pilot study from 2014 to 2018 centered on the Native Hawaiian traditional practice of “hale mua” (men’s house) to address colorectal cancer-related health disparities among Native Hawaiian men [15]. The study used a peer-led intervention model in which group discussions and educational sessions were facilitated by kāne (Native Hawaiian men) volunteers and Native Hawaiian physicians. Discussions were held at community-based venues not affiliated with any healthcare organization. Education materials and curricula were developed on the basis of input from Native Hawaiian physicians and an expert consultant on Hawaiian culture. They were then modified using an iterative process based on input from kāne committee members, discussion group facilitators, and study participants.

Group discussions focused on the risks, common causes, and prevention of colorectal cancer using a motivational interviewing approach. Participants over 50 years old who opted into completing a FIT were given a FIT kit designed specifically for kāne. Overall, there were 232 participants who attended 21 sessions on colorectal cancer screening, of which 64% (149/232) were over age 50. Survey data showed that 31% (46/149) of participants over age 50 had not discussed their colon health or screening with their doctor. Almost all participants over age 50 (92%; 137/149) improved their knowledge from the sessions and 76% (113/149) agreed to complete a FIT test. Final screening results showed that 79% (117/149) of participants over age 50 were up to date with colorectal cancer screening.

#### 4.1.2. Multicomponent Intervention

Tolma et al. (2018) did a formative evaluation to determine the feasibility and early impact of an intervention promoting mammography screening among AI/AN called the Native Women’s Health Project (NWHP) [16]. The NWHP took place in a tribal clinic and the surrounding Native American community southeast of Oklahoma City between June and December 2014.

The study included both clinic- and community-based components that promoted mammography screening through multiple system levels, including individual, family/social support, organizational/practice, and community/environmental levels. The clinic-based component included a patient-doctor discussion on mammography screening (informed by what decision stage the patient was in), a mammography brochure and poster, a follow-up letter, and a flowchart used by the doctor when advising patients. The community-based component included six 90-min intergenerational discussion groups and a congratulatory gift upon completion of a mammogram.

None of the participants had a mammogram in the two years prior to the study. The study showed moderate success with over half of 21 participants (52%; *n* = 11; 95% CI = 30% to 74%) undergoing mammography within six months after the end of the intervention. Additionally, nearly a third of 20 participants (30%; *n* = 6; 95% CI = 15% to 52%) improved their intention to undergo mammography during the intervention. Qualitative analysis showed that women better understood the importance of being aware of breast changes after the end of the intervention.

Although the early impact of this program showed some promise, one limitation is the lack of generalizability, due to a small sample size and moderate response rate. Replication of the study is needed with a larger sample and longer implementation time.

### 4.2. Preference and Acceptability of HPV Self-Sampling

Three studies showed an acceptability and preference for HPV self-sampling, including studies targeting Māori women in New Zealand [14], Hopi women in Arizona [18], and FN women in Ontario (see Table 4) [17]. Doing self-sampling in one’s own home may allow more rural and Indigenous people to participate in cervical screening by removing the need for clinicians to conduct the test. It may also make some feel more comfortable due to increased privacy and personal control [19]. While some women were worried about whether a self-collected sample would be as accurate as a clinician -collected sample [20], HPV self-sampling has been shown to have comparable sensitivity and specificity to clinician-sampling [21,22,23].

There was high variability in uptake of HPV self-sampling and clinician-sampling in a Canadian community RCT by Zehbe et al. (2016). The range of uptake was 0.0% to 62.1% in self-sampling communities and 0.0% to 47.1% in clinician-sampling communities. Initial uptake of self-sampling was also 1.4-fold higher compared to clinician-sampling. Providing HPV self-sampling combined with community engagement and culturally sensitive education may be a feasible option for under-screened FN women in Canada (Zehbe et al., 2017). However, more evidence is needed to determine logistics and cost-effectiveness of adding an HPV self-sampling option to a population based cervical cancer screening program.

## 5. Conclusions

This review began with no *a priori* assumptions about the importance of intervention factors, with the exception of respectful engagement with Indigenous community leaders. The investigation of studies that successfully increased screening rates or knowledge, attitude or intent to screen among Indigenous people sought information on these key intervention characteristics: how they were developed, how well the intervention fit the needs and preferences of the target communities, the level of engagement that occurred, the preparation/training required, and communication methods (see Table 5).

Indigenous cancer screening interventions were identified that were effective and feasible for specific Indigenous populations and specific cancer screening programs. Twelve interventions met the inclusion criteria for this review. The included studies were effective in increasing cancer screening participation rates or showed promise for this outcome based on improvement of knowledge, attitude, or intent to screen for breast, colorectal, or cervical cancer. Intervention strategies included both text and telephone reminder systems, opportunistic screening, mobile screening, PDSA cycles, peer-led education, HPV self-sampling methods, and mailed FIT kits. Target populations included AN/AI and Native Hawaiian people from the United States, FN and Métis people from Canada, Māori, Pacific, and Asian people from New Zealand, and Torres Strait Islanders from Australia (see Table 6).

Key components found in most of these studies included engagement with Indigenous leaders or tribal healthcare groups which supported reciprocal relationships between researchers and clinicians and their personnel who were identified as community-based supports with the targeted population. For example, having direct contact with the targeted population rather than just administering a survey seemed to be identified as a factor for improved participation in the screening. About half of the interventions indicated use of community coordinators to assist with implementation and outreach, which may have helped establish trust. The use of community coordinators who spoke to participants in their native languages was also indicated in several studies—this may have promoted a better understanding of the screening program by overcoming language and cultural barriers. All interventions were designed around overcoming certain barriers to screening, and several specifically indicated using knowledge of community preferences in designing the intervention. Multiple, culturally appropriate strategies can overcome barriers to screening beyond just increasing knowledge and awareness.

Critical to the success of any health promotion or cancer prevention efforts are co-development by leaders from the Indigenous populations and screening programs to design, implement, and evaluate community-based participatory interventions prior to a full roll out. These efforts can build on the key characteristics of positive interventions and plan for ongoing evaluation to provide feedback on the longer-term impact of these interventions. Cancer screening is part of organized health care so challenges to participation will likely remain until pervasive larger issues are overcome, including lack of trust in health care providers and organizations, racism in health care delivery, complex and sometimes fragmented health care delivery and social determinants of health that led to health disparities.

## Figures and Tables

**Table 1:** Databases searched and search terms.

Search Terms	
Databases searched	Native Health Database, MEDLINE (Ovid), Cochrane Library, PsycINFO, PubMed, PubMed Central, CINAHL, MEDLINE (Ebsco), Psychology & Behavioral Sciences Collection, and HealthSTAR
Population-specific terms/phrases used	Aboriginal, Indigenous, Inuit, First Nations, Métis, native people, native Canadian, Māori, and Native American
Disease-specific terms/phrases used	Breast cancer screening, cervical cancer screening, colorectal cancer screening, early detection of cancer, mammogram, mammography, pap, pap smear, fecal immunochemical test, faecal immunochemical test, fecal occult blood test, faecal occult blood test, breast, cervix, colon, rectum, cancer, carcinoma, neoplasm, tumour, oncology, and mass screening

**Table 2:** Inclusion and exclusion criteria for the selection of articles.

Articles	Inclusion Criteria	Exclusion Criteria
Population	Study participants were from urban or rural Indigenous populations in Canada (FNIM), the United States (American Indian, Alaskan Native), Australia (Torres Straight Islanders, or New Zealand (Māori).	The study didn’t focus on Indigenous populations or have a separate assessment of Indigenous groups.
Intervention	The study included a program, practice, activity, pilot, strategy, or tool focused on cervical, colorectal, or breast cancer screening. The study’s primary goal was to improve cancer screening rates or knowledge, attitudes, or intention to screen. The intervention was feasible within the context of the study’s target population and may be applicable to other health settings	The study focused on screening programs for diseases other than cervical, colorectal, or breast cancer. The study focused on identifying health disparities or risk factors for cancer incidence or mortality (i.e., not actionable). The study was not feasible or applicable to other health settings.
Outcome	The study increased cancer screening participation rates in an Indigenous population. The study showed promise based on improving process indicators of the outcome (e.g., knowledge, attitude, or intent to screen).	The study focused on data regarding health disparities or risk factors for cancer incidence or mortality (i.e., not actionable)
Other	The article was written in English. The article was published between 1 January 2014 and 12 March 2021.	The article was not written in English. The article was published prior to 1 January 2014.

**Table 3:** Interventions that increased screening participation.

Citation	Cancer Screening Type	Setting	Sample	Study Design & Intervention	Outcome: Screening Participation
Muller et al., 2017 [7]	Colorectal cancer	Anchorage, Alaska	2386 Alaskan Native and Native American men and women, aged 40 to 75 years	RCT: Addition of text message reminders to existing electronic reminders	Age groups: Age 40–49: 24% increase Age 50–75: 42% increase All ages: 30% * increase
Sandiford et al., 2019 [8]	Colorectal cancer	New Zealand	7601 Māori, Pacific, and Asian men and women, aged 50 to 74 years	RCT: Addition of a telephone call to existing letter reminders	Ethnic groups: Māori: 5.2% * increase Pacific: 3.6% * increase Asian: 0.7% increase
MacDonald et al., 2021 [9]	Cervical Cancer	Northland, New Zealand	931 Māori women, aged 25–69 years	RCT: Addition of HPV self-test	Standard care: 21.8% screened HPV self-test: 59.0% screened (2.8 * times higher)
Haverkamp et al., 2020 [10]	Colorectal cancer	Southwest United States	1288 Alaskan Natives and American Indians	RCT: Addition of mailed FIT kits or mailed FIT kits plus follow-up outreach by phone/home visit	Standard care: 6.4% screened Mailed FIT kit: 16.9% * screened Mailed FIT kit + outreach: 18.8% * screened
Chow et al., 2020 [11]	Breast, cervical, colorectal cancer	Wequedong Lodge in Ontario	First Nations men and women, aged 50–74 years (breast and colorectal) and 21–69 years (cervical)	Pilot study: Education and opportunistic cancer screening	Year: 2014–2015: 62% increase 2015–2016: 68% increase
Mema et al., 2017 [5]	Cervical and colorectal cancer	Northern Alberta	First Nations, Métis and Hutterite women, aged 50 to 74 years	Pilot study: Integration of cervical and colorectal screening with the Screen Test mobile mammography program	Cancer type: Total screened: Usual Practice (Screen-Test mobile mammography) Cervical: 10.1% Colorectal: 10.9% Integrated Approach (Screen-Test-EACS) Cervical: 27.5% Colorectal: 22.5%
Dorrington et al., 2015 [12]	Cervical cancer	Australia	Aboriginal and Torres Strait Islander women, aged 18 to 70 years	PDSA Cycles: translational research and continuous quality improvement	Year: 2012: 40% * increase

* Statistically significant (*p* value < 0.05), RCT = randomized controlled trial, EACS = Enhanced Access to Colorectal and Cervical Screening, PDSA = Plan-Do-Study-Act.

**Table 4:** Interventions that improved knowledge, attitude, or intention to screen.

Citation	Cancer Type	Setting	Sample	Study Design & Intervention	Outcome
Cassel et al., 2020 [15]	Colorectal	Hawaii, USA	378 Native Hawaiian men, aged 18+, with focus on ages 50+ for use of FIT	Peer-led model: group discussions and educational sessions.	92% improved their knowledge about colon health and 76% agreed to complete a FIT.
Tolma et al., 2018 [16]	Breast	Oklahoma City, USA	21 American Indian/ Alaska Native women, aged 52–74 years	Formative evaluation: Multicomponent (clinic and community components)	30% improved their intention to do a mammogram. 52% had a mammogram by six months post-intervention.
Adcock et al., 2019 [14]	Cervical	New Zealand	503 Māori women, aged 25+ years and 17 healthcare providers	Mixed qualitative and quantitative: Focus groups/interviews, survey	75% of Māori survey participants reported being likely/very likely to do an HPV self-test
Zehbe et al., 2016 [17]	Cervical	NW Ontario, Canada	834 First Nations Women, aged 25–69 years	Community RCT: HPV self-sampling (Arm A) and Pap testing (Arm B)	Initial uptake in Arm A was 1.4-fold higher than arm B Range of uptake: Arm A: 0.0% to 62.1% Arm B: 0.0% to 47.1%.
Winer et al., 2016 [18]	Cervical	NE Arizona, USA	329 Hopi women, aged 21–65 years	Cross-sectional: Recruitment within community to complete HPV self-sampling	62% reported a preference for self-sampling

**Table 5:** Descriptive Table of Key Characteristics of the Interventions.

Citation	Intervention Development	Community Needs and Preferences	Community Engagement	Workforce Preparation	Communication Methods
Muller et al., 2017 [7]	Developed in coordination with SCF, a tribally owned and operated health care organization.	Previous survey findings showed the majority of customer-owners over 50 used text messaging.	Text message content was developed with input from SCF customer-owners and tribal leadership.	The intervention was integrated into an existing SCF program.	The intervention group received up to 3 text messages sent 1 month apart.
Sandiford et al., 2019 [8]	Follow-up to an existing Bowel Screening Pilot using mailed invitation and reminder letters.	Patient and cultural barriers	During telephone calls, community coordinators sought to remove any barriers to screening, such as how to perform the test.	The callers’ script was reviewed by health literacy experts.	All non-respondents were sent reminder letters. The intervention group also received 3+ phone calls over 4 weeks. Community coordinators spoke with participants in their native languages.
MacDonald et al., 2021 [9]	Follow-up to a survey showing high acceptability for HPV self-testing among Māori women.	Patient and cultural barriers	The study was under-taken in partnership with primary care and the Northland District Health.	Clinic staff were given an educational update on HPV, informed consent, and the HPV self-test.	Text, email, letter, and phone calls from clinics and outreach by kaiāwhina.
Haverkamp et al., 2020 [10]	Developed in partnership with 3 tribally operated health facilities that participated in study.	Patient and structural barriers †	American Indian CHRs contacted intervention nonrespondents to discuss the importance of CRC screening and how to use the FIT kit.	Clinic admin and staff were informed about the study and CHRs were educated about screening recommendations and intervention protocol.	FIT kits were mailed to intervention groups and CHRs provided outreach (i.e., phone calls and home visits).
Chow et al., 2020 [11]	Developed in partnership with the Wequedong Lodge, TBRHSC, the Nishnawbe Aski Nation Chiefs Assembly, and CCO’s. Indigenous Cancer Care Unit.	Geographic, transportation, and cultural barriers	Cancer screening education and opportunistic screening was provided for those staying at the lodge (mostly from rural FN populations).	Community chiefs and physicians were notified about the program and given information about program logistics and patient follow-up.	A FN liaison spoke with clients in their native language. A FN-specific education toolkit was used during appointments.
Mema et al., 2017 [5]	Provision of ‘one stop shop’ cancer screening services in many communities, including FN.	Geographical barriers—communities were chosen based on their need for cancer screening services using a readiness assessment tool.	Leverage existing relationships with mobile mamography service.	Local clinical staff provided Pap and FIT tests.	Recall letters were sent to all clients who had participated in Screen Test in the past and were due for breast cancer screening.
Dorrington et al., 2015 [12]	Interventions were designed based on PDSA cycles and tested for cultural acceptability with the ACCHS Women’s Group.	Patient barriers	Client surveys and focus groups with stakeholders	The Social Health Team was educated on women’s preventative health and cervical cancer screening to faciliate discussions with ACCHS clients. HCPs were educated on how to use a data collection tool for Pap smears.	Promotional material was used to raise awareness of cervical screening. A reminder letter was updated to include culturally appropriate cervical cancer screening information.
Cassel et al., 2020 [15]	A peer-led intervention facilitated by kāne and Native Hawaiian physicians.	CRC health dispartities among Native Hawaiian men	Discussions about CRC were held at community-based venues and participants were given a FIT kit.	Education materials and curricula were developed by Native Hawaiian physicans and modified based on community feedback.	21 community sessions on CRC screening.
Tolma et al., (2018) [16]	Formative evaluation to determine the feasibility and early impact of a CBPR intervention.	Geographical disparties	Clinic and community-based components on multiple system levels.	Evaluation planning based on years of formative research in the community.	Communication with HCP, discussion groups, and a congratulatory gift.
Adcock et al., 2019 [11]	This study explored the potential acceptability of an intervention.	Desire for bodily autonomy (privacy, control over ones body)	Focus groups, interviews, and surveys with never/underscreened Māori women.	Not addressed	CBRs recruited Māori women for interviews and focus groups. Participants surveyed up to 10 Māori female peers.
Zehbe et al., 2016 [17]	Designed with 11 FN partner communities using a PAR framework.	Geographic and cultural barriers	Interviews and focus groups with HCPs and women living on reserves about CC screening barriers.	CBRAs invited women to participate after an educational event and other recruitment strategies.	CBRAs facilitated screening implementation and data collection. Participants were asked how they wanted to be contact if they had a positive HPV test result.
Winer et al., 2016 [18]	Designed with input from Hopi tribal partners, local project staff, and community advisors.	Patient barriers	In-person community recruitment events	Not addressed	Recruitment flyers and informational brochures were given at community events, door-to-door health education campaigns, and tribal radio announcements. HPV test results were communicated by letter or telephone, based on preference.

SCF = Southcentral Foundation, Customer-owners = SCF patients, CHR = community health representative, TBRHSC = Thunder Bay Regional Health Sciences Centre, CCO = Cancer Care Ontario, ACCHS = Aboriginal Community Controlled Health Service, HCP = Health Care Provider, kāne = Native Hawaiian men, kaiāwhina = non-clinical community Māori health workers, PAR = participatory action research, CBPR = community-based participatory research, CBRAs = community-based research assistants † Patient and structural barriers may include geographic isolation, lack of transportation, not having a regular HCP, failure of HCP to recommend screening, lack of a clinical tracking/reminder system, embarrassment, privacy concerns, distrust of the health care system, and insufficient knowledge about screening.

**Table 6:** Final summary table of included studies.

Studies	Citations	Study Design	Cancer Screening Types	Sample	Outcomes
**Seven studies reported an increase in cancer screening participation**	Muller, 2017 [7]	RCT	CRC	Alaskan Native; Native American	*Age group:* 40–49: 24% increase 50–75: 42% increase All ages: 30% * increase
	Sandiford, 2019 [8]			Māori, Pacific	*Ethnic group:* Māori: 5.2% * increase Pacific: 3.6% * increase Asian: 0.7% increase
	MacDonald et al., 2021 [9]		CC	Māori	*Standard care:* 21.8% screened *HPV Self-sampling:* 59.0% screened (2.8 * times higher)
	Haverkamp et al., 2020 [10]		CRC	Alaskan Native/American Indian	*Standard care:* 6.4% screened *Mailed FIT kit:* 16.9% * screened *Mailed FIT kit + outreach:* 18.8% * screened
	Chow, 2020 [11]	Pilot	CRC, CC, BC	First Nations	*Year:* 2014–2015: 62% increase 2015–2016: 68% increase
	Mema, 2017 [5]		CC, BC	First Nations, Métis, Hutterite	*Total screened:* Usual Practice (Screen-Test mobile mammography) Cervical: 10.1% Colorectal: 10.9% Integrated Approach (Screen-Test EACS) Cervical: 27.5% Colorectal: 22.5%
	Dorrington, 2015 [12]	PDSA cycles	CC	Torres Strait Islander	*Year:* 2012: 40% * increase
**Five studies improved knowledge, attitude, or intent to screen**	Cassel, 2020 [15]	Peer-led	CC	Native Hawaiian	92% improved their knowledge 76% agreed to complete a FIT
	Tolma, 2018 [16]	Multi-level	BC	Native American	30% improved their intent to screen 52% had a mammogram by 6 months post-intervention.
	Zehbe, 2016 [17]	RCT	CC (HPV self-sampling)	First Nations	Initial uptake in HPV self-sampling was 1.4-fold higher than clinician-sampling
	Adcock, 2019 [14]	Mixed		Māori	75% reported being likely/very likely to do an HPV self-test
	Winer, 2016 [18]	Cross-sectional		Hopi	62% reported a preference for HPV self-sampling

BC = breast cancer; CRC = colorectal cancer; CC = cervical cancer, RCT = randomized controlled trial; PDSA = Plan-Do-Study-Act, EACS = Enhanced Access to Colorectal and Cervical Screening, * Statistically significant (*p* value < 0.05).

## Data Availability

Data sharing not applicable.
